# Supplementary material for: Association of inflammation-related markers and diabetic retinopathy severity in the aqueous humor, but not serum of type 2 diabetic patients
Source: PLoS One. 2023 Oct 26;18(10):e0293139. doi: 10.1371/journal.pone.0293139 (PMC10602301; doi:10.1371/journal.pone.0293139)
Supplement: S1 Checklist — (DOCX) [file pone.0293139.s002.docx]

STROBE Statement—checklist of items that should be included in reports of observational studies

|  | Item No. | Recommendation | Page  No. | Relevant text from manuscript |
| --- | --- | --- | --- | --- |
| **Title and abstract** | 1 | (*a*) Indicate the study’s design with a commonly used term in the title or the abstract | 2 | “In this retrospective, observational pilot study …” |
|  |  | (*b*) Provide in the abstract an informative and balanced summary of what was done and what was found | 2 |  |
| Introduction | | | |  |
| Background/rationale | 2 | Explain the scientific background and rationale for the investigation being reported | 3-4 |  |
| Objectives | 3 | State specific objectives, including any prespecified hypotheses | 4 | “In this pilot study, we aimed to determine…” |
| Methods | | | |  |
| Study design | 4 | Present key elements of study design early in the paper | 4-6 |  |
| Setting | 5 | Describe the setting, locations, and relevant dates, including periods of recruitment, exposure, follow-up, and data collection | 4-5 | “This retrospective study drew (…). Samples of AH were obtained at the beginning of the surgery in the Clinic for Vitreoretinal Diseases, Berner Augenklinik, Bern, Switzerland. (…) Serum and AH samples were obtained between 2015 and 2022 (…) which was conducted between June and July 2022.” |
| Participants | 6 | (*a*) *Cohort study*—Give the eligibility criteria, and the sources and methods of selection of participants. Describe methods of follow-up  *Case-control study*—Give the eligibility criteria, and the sources and methods of case ascertainment and control selection. Give the rationale for the choice of cases and controls  *Cross-sectional study*—Give the eligibility criteria, and the sources and methods of selection of participants | 4-5 | “This retrospective study drew on 42 patients with T2DM with or without DR and 19 healthy individuals without any known systemic or ocular disease who underwent cataract surgery. (…) Exclusion criteria included (…)” |
|  |  | (*b*) *Cohort study*—For matched studies, give matching criteria and number of exposed and unexposed  *Case-control study*—For matched studies, give matching criteria and the number of controls per case |  | N/A |
| Variables | 7 | Clearly define all outcomes, exposures, predictors, potential confounders, and effect modifiers. Give diagnostic criteria, if applicable | 6 | “The following clinical variables have been measured (…)” |
| Data sources/ measurement | 8* | For each variable of interest, give sources of data and details of methods of assessment (measurement). Describe comparability of assessment methods if there is more than one group | 5 | “All samples were analyzed using multiplex immunoassay kits based on Luminex MAP technology, as previously described” |
| Bias | 9 | Describe any efforts to address potential sources of bias | 6 | “To minimize impact of bias we included (…)” |
| Study size | 10 | Explain how the study size was arrived at |  | N/A (pilot study) |

Continued on next page

| Quantitative variables | 11 | Explain how quantitative variables were handled in the analyses. If applicable, describe which groupings were chosen and why | 6 |  |
| --- | --- | --- | --- | --- |
| Statistical methods | 12 | (*a*) Describe all statistical methods, including those used to control for confounding | 6-7 |  |
|  |  | (*b*) Describe any methods used to examine subgroups and interactions | 6-7 |  |
|  |  | (*c*) Explain how missing data were addressed | 8 | “Serum RBP-4 and IL-6 ranged above the detection limit in only 36% and 13% of samples, respectively, and were thus excluded from the statistical analyses. All the other target proteins ranged within the limits of detection of the assay in all samples.” |
|  |  | (*d*) *Cohort study*—If applicable, explain how loss to follow-up was addressed  *Case-control study*—If applicable, explain how matching of cases and controls was addressed  *Cross-sectional study*—If applicable, describe analytical methods taking account of sampling strategy |  | N/A |
|  |  | (*e*) Describe any sensitivity analyses |  | N/A |
| Results | | | | |
| Participants | 13* | (a) Report numbers of individuals at each stage of study—eg numbers potentially eligible, examined for eligibility, confirmed eligible, included in the study, completing follow-up, and analysed |  | N/A (Since this is a cross sectional pilot study, we included a limited number of samples) |
|  |  | (b) Give reasons for non-participation at each stage |  | N/A |
|  |  | (c) Consider use of a flow diagram |  | N/A |
| Descriptive data | 14* | (a) Give characteristics of study participants (eg demographic, clinical, social) and information on exposures and potential confounders | 7 |  |
|  |  | (b) Indicate number of participants with missing data for each variable of interest | 7 |  |
|  |  | (c) *Cohort study*—Summarise follow-up time (eg, average and total amount) |  | N/A |
| Outcome data | 15* | *Cohort study*—Report numbers of outcome events or summary measures over time |  | N/A |
|  |  | *Case-control study—*Report numbers in each exposure category, or summary measures of exposure |  | N/A |
|  |  | *Cross-sectional study—*Report numbers of outcome events or summary measures |  | See figures 1, 2A and 2B |
| Main results | 16 | (*a*) Give unadjusted estimates and, if applicable, confounder-adjusted estimates and their precision (eg, 95% confidence interval). Make clear which confounders were adjusted for and why they were included |  | N/A (see statistics section) |
|  |  | (*b*) Report category boundaries when continuous variables were categorized |  | N/A |
|  |  | (*c*) If relevant, consider translating estimates of relative risk into absolute risk for a meaningful time period |  | N/A |

Continued on next page

| Other analyses | 17 | Report other analyses done—eg analyses of subgroups and interactions, and sensitivity analyses |  | See results section |
| --- | --- | --- | --- | --- |
| Discussion | | | | |
| Key results | 18 | Summarise key results with reference to study objectives | 12-16 |  |
| Limitations | 19 | Discuss limitations of the study, taking into account sources of potential bias or imprecision. Discuss both direction and magnitude of any potential bias | 15 | “The limitations include (…)” |
| Interpretation | 20 | Give a cautious overall interpretation of results considering objectives, limitations, multiplicity of analyses, results from similar studies, and other relevant evidence |  | See discussion section |
| Generalisability | 21 | Discuss the generalisability (external validity) of the study results | 15 | “Consequently, the results of this pilot study need to be corroborated with a future prospective study including a larger number of patients.” |
| Other information | |  | | |
| Funding | 22 | Give the source of funding and the role of the funders for the present study and, if applicable, for the original study on which the present article is based | 1 | “Founding information: STIFTUNG LINDENHOF 17-05-F» |

*Give information separately for cases and controls in case-control studies and, if applicable, for exposed and unexposed groups in cohort and cross-sectional studies.

**Note:** An Explanation and Elaboration article discusses each checklist item and gives methodological background and published examples of transparent reporting. The STROBE checklist is best used in conjunction with this article (freely available on the Web sites of PLoS Medicine at http://www.plosmedicine.org/, Annals of Internal Medicine at http://www.annals.org/, and Epidemiology at http://www.epidem.com/). Information on the STROBE Initiative is available at www.strobe-statement.org.
