# Supplementary material for: Association of inflammation-related markers and diabetic retinopathy severity in the aqueous humor, but not serum of type 2 diabetic patients
Source: PLoS One. 2023 Oct 26;18(10):e0293139. doi: 10.1371/journal.pone.0293139 (PMC10602301; doi:10.1371/journal.pone.0293139)
Supplement: Attachment — Submitted filename: PONE-D-23-14800 Response to reviewers 280923.docx [file pone.0293139.s003.docx]

**Pone-D-23-14800 Response to the Editor and reviewer**

We thank for the opportunity to revise our paper. The manuscript has been revised and text and tables habe been modified to meet PLOS ONE’s style requirements. Following changes have been made to meet the expectations of editor and reviewer:

**Editor:**
Thank you for stating the following in the Competing Interests section:

"Justus G. Garweg acts as an advisor and speaker for several pharmaceutical companies (AbbVie, Roche, Bayer, and Novartis) and participates in several international industry-sponsored clinical studies. All the authors have declared that no competing interests exist. "

Please confirm that this does not alter your adherence to all PLOS ONE policies on sharing data and materials, by including the following statement: "This does not alter our adherence to  PLOS ONE policies on sharing data and materials.” (as detailed online in our guide for authors <http://journals.plos.org/plosone/s/competing-interests>).  If there are restrictions on sharing of data and/or materials, please state these. Please note that we cannot proceed with consideration of your article until this information has been declared.

Please include your updated Competing Interests statement in your cover letter; we will change the online submission form on your behalf.

**Response:**

We have updated the Competing Interests statement and reported it in the cover letter.

**Editor:**
In your Data Availability statement, you have not specified where the minimal data set underlying the results described in your manuscript can be found. PLOS defines a study's minimal data set as the underlying data used to reach the conclusions drawn in the manuscript and any additional data required to replicate the reported study findings in their entirety. All PLOS journals require that the minimal data set be made fully available. For more information about our data policy, please see <http://journals.plos.org/plosone/s/data-availability>.

Upon re-submitting your revised manuscript, please upload your study’s minimal underlying data set as either Supporting Information files or to a stable, public repository and include the relevant URLs, DOIs, or accession numbers within your revised cover letter. For a list of acceptable repositories, please see <http://journals.plos.org/plosone/s/data-availability#loc-recommended-repositories>. Any potentially identifying patient information must be fully anonymized.

Important: If there are ethical or legal restrictions to sharing your data publicly, please explain these restrictions in detail. Please see our guidelines for more information on what we consider unacceptable restrictions to publicly sharing data: <http://journals.plos.org/plosone/s/data-availability#loc-unacceptable-data-access-restrictions>. Note that it is not acceptable for the authors to be the sole named individuals responsible for ensuring data access.

We will update your Data Availability statement to reflect the information you provide in your cover letter.

**Response:**
Our data set has been included in a Supporting Information file and is uploaded with the resubmission.

**Editor:**
Please include your full ethics statement in the ‘Methods’ section of your manuscript file. In your statement, please include the full name of the IRB or ethics committee who approved or waived your study, as well as whether or not you obtained informed written or verbal consent. If consent was waived for your study, please include this information in your statement as well.

**Response:**
This information can be found in the methods section of the manuscript, lines 107-112

5. Please review your reference list to ensure that it is complete and correct. If you have cited papers that have been retracted, please include the rationale for doing so in the manuscript text, or remove these references and replace them with relevant current references. Any changes to the reference list should be mentioned in the rebuttal letter that accompanies your revised manuscript. If you need to cite a retracted article, indicate the article’s retracted status in the References list and also include a citation and full reference for the retraction notice.

**Response:**
The reference list has been revised and no changes were made. No retracted papers have been cited.

**Additional Editor Comments:**

Excellent manuscript. All concerns are minor.
In addition to reviewer 1. On-Line 129, it states that “Two researchers extracted the clinical data and did a double coding to avoid rater bias.” Were the proteomic measurements also done in a blinded manner and then sorted into the groups for statistical analysis? If it was, please include it in the methods.

**Response:**
We would like to thank the editor for his favourable rating and for the very relevant comment. It was added in the materials and methods section, lines 124-126.


**Reviewers' comments:**

Reviewer #1: Dear Author
I had the privilege of reviewing your manuscript.
Taking into account the striking difference observed in normal patients versus diabetic patients in the aqueous humor levels of the biomarkers, what are the subsequent studies devised by your group to try to address the increased secretion of those inflammatory biomarkers ?
Do you think intraocular steroid therapy may decrease the secretion of those biomarkers and maybe delay the retinopathy progression?
A new study addressing patients injected with intravitreal slow release dexamethasone may show some promise in controlling that expression?
Those are subjects worth discussing in the manuscript.
Best

**Response:**
We thank very deeply the reviewer for his excellent feedback and for taking the time to review our manuscript. We sincerely appreciate this review. We found the comments very insightful and the raised topics were added in the discussion, lines 381-4: “Based on our observations, we have initiated a prospective study with more power to further investigate the robustness of these findings and to assess their potential role in type 1 DM. If confirmed, we wish to investigate in a follow up analysis the impact of treatment, i.e. intravitreal anti-VAGF and corticosteroid therapy”.
